# Integrated analysis of how gender and body weight affect the intestinal microbial diversity of *Gymnocypris chilianensis*

**DOI:** 10.1038/s41598-023-35600-y

**Published:** 2023-05-31

**Authors:** Zhongmeng Zhao, Han Zhao, Lu Zhang, Zhipeng Huang, Hongyu Ke, Ya Liu, Yuanliang Duan, Huadong Li, Xiongyan Wang, Qiang Li

**Affiliations:** 1 Present Address: Fisheries Institute, Sichuan Academy of Agricultural Sciences, 1611 Xiyuan Avenue, Chengdu, Sichuan China; 2 Sichuan Water Conservancy Vocational College, Chongzhou, Sichuan China

**Keywords:** Microbial ecology, Microbiome

## Abstract

Intestinal microorganisms that living in the mucosa and contents of the gastrointestinal tract of animals, have close links with their hosts over a long evolutionary history. The community structure of the fish intestinal microbiota is associated with food, living environment, and the growth stage. To screen for potential probiotics that can be used for regulating breeding behaviors, this study focused on the diversity of fish intestinal microorganisms. This study aimed to investigate the effects of sex and body weight on the intestinal microbial diversity of *Gymnocypris chilianensis* in the wild. The results showed that the significant high diversity and richness of intestinal microbiota were fould in heavier individuals, and males. The dominant bacterial phyla of *G. chilianensis* were Proteobacteria, Firmicutes, and Bacteroidetes. In addition, the abundance of Firmicutes varied significantly among different body weights. The genus profile revealed that small individuals were dominated by *Weissella*, while females were dominated by *Aeromonas*, and both large individuals and males were dominated by other genera. Phylogenetic relationships and UPGMA clustering analysis showed significant differences among the groups. In general, the two main factors that have an effect on the intestinal microbiota diversity of wild *G. chilianensis* are sex and body weight.

## Introduction

*Gymnocypris chilianensis* a freshwater fish of the Cyprinidae family and *Gymnocypris* genus, which is only distributed in the three inland river basins of the Hexi Corridor in the Gansu Province, known as the Heihe River, the Shule River, and the Shiyang River^1^. Therefore, It is an important indigenous economic fish and a key protected wild fish in this province^2^.

These fish reach sexual maturity at approximately 4 years of age and reproduce from April to May each year. In recent years, due to the comprehensive impact of natural and human factors (e.g., the imbalance of the ecological environment in the inland river basin, the construction of water conservancy projects, and overfishing), the natural distribution of this fish has been greatly reduced, as has the wild population^3^.

In recent years, the development of ‘omics’ technologies is increasing research on the structure and function of the gastro intestinal microbiota of fish^4^. In the normal physiological state, the intestinal microbiota maintains a dynamic balance through its interactions between the microorganisms and the host, which jointly affect the host’s nutritional immune performance, physiological state, diseases, and other biological processes^5^. Previous studies have shown that many intestinal microbiota can secrete digestive enzymes to decompose starch and cellulose in feed to promote nutrient absorption. Many microbes are also known to promote the development of the host's immune system, thereby improving overall health^6^. Therefore, it is significant to maintaining the homeostasis of intestinal microbiota for improving the health, the growth, and development of the host.

Multiple factors such as the host genotype, sex, environment, growth stage, and nutrient availability, have effects on the homeostasis of the intestinal microbiota^7–9^. These factors have been extensively demonstrated in study of mammals, and more related studies have been reported in fish and crustaceans^9–11^. In addition, there exists an increasing demand for the artificial reproduction of *G. chilianensis*, which is a key economic fish in Gansu Province. Considering the importance of intestinal microbiota in fish health, this study measured the intestinal microbiota of *G. chilianensis* of different weights and sexes from the Shule River via 16S rRNA gene sequencing. The sex-dependent effect of its intestinal microbiota can provide a theoretical basis for the successful reproduction of *G. chilianensis*. Furthermore, the difference between body weights should be accounted for the screening of intestinal probiotics during fish growth and the study of probiotic preparations in the aquaculture industry.

## Results

### Sequencing characteristics of Intestinal microbiota in *G. chilianensis*

**Figure 1:**
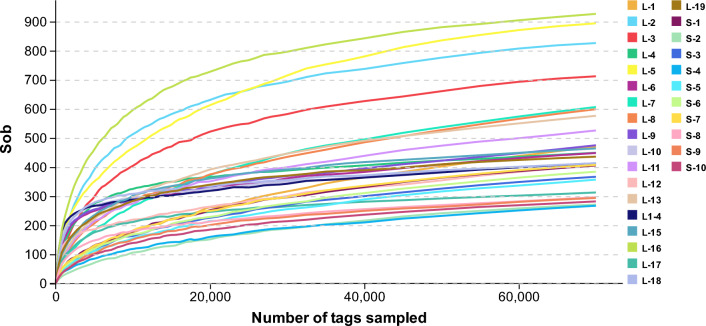
Dilution curves for the Sob index of the MiSeq sequences of the 16S rRNA gene from different body weight samples. The colors of lines represent fish samples. The horizontal and vertical coordinates reflects the number of extracted tags and the corresponding diversity index value calculated when the corresponding number of tags is extracted, renectively. S: small fish, L: large fish.

We obtained 3,661,658 clean tags from the 29 fish intestines. A remaining 3,508,043 effective tags were obtained after removing the chimeric tags detected in the clustering analogy pair. We calculated the number of operational taxonomical units (OTUs) and the coverage was found to be over 99.70% for each sample (Table 1). The rank abundance curves of all samples were obtained based on the OTU abundance of each sample and its ranking. Figure 1 reflects the richness and evenness of the species composition of each sample.

**Table 1:** Number of clean Tags, effective tags, OTUs, and good’s coverage for 16S rRNA libraries of different fish samplings.

Samplings	Clean tags	Effective tags	OTUs	Good’s coverage (%)
F-1(L-1)	134604	128561	574	99.72
F-2(L-2)	123693	118411	891	99.78
F-3(L-3)	123154	118493	772	99.78
F-4(L-4)	118977	116866	491	99.88
F-5(L-5)	130555	117468	895	99.82
F-6(L-6)	135447	132829	527	99.82
F-7(L-7)	134258	124283	673	99.74
F-8(L-8)	132937	127713	700	99.70
F-9(L-9)	130743	128505	559	99.79
F-10(L-10)	119490	116865	458	99.87
M-1(L-11)	119793	116240	594	99.77
M-2(L-12)	131280	129493	505	99.77
M-3(L-13)	128015	119774	627	99.77
M-4(L-14)	121815	120529	465	99.87
M-5(L-15)	121383	116606	495	99.88
M-6(L-16)	122782	116656	954	99.82
M-7(L-17)	129205	127239	348	99.91
M-8(L-18)	117853	116762	451	99.89
M-9(L-19)	132180	128007	474	99.89
S-1	127680	116920	439	99.83
S-2	125464	122031	323	99.85
S-3	118948	109482	396	99.83
S-4	132187	122986	309	99.86
S-5	123503	116236	404	99.82
S-6	128821	118168	421	99.82
S-7	123413	113193	442	99.82
S-8	122335	120156	334	99.89
S-9	126675	125598	338	99.89
S-10	124468	121973	328	99.88

*OTU* Operational taxonomical unit, *S* Small fish, *L* Large fish, *F* Female fish, *M* Male fish.

### Intestinal microbial communities in *G*. *chilianensis* with different body weights and genders

The 29 samples were divided into two groups according to body weight; 19 fish weighing more than 300 g were considered large fish, and 10 fish weighing less than 300 g were considered small fish. *G. chilianensis* generally grow to about 300 g when it reaches sexual maturity (i.e., 4-year-old). In addition, the large 19 fish were divided into the male and female groups by their gonads. There were 10 fish in the male group which labeled by “M” in Table 1, and 9 fish in the female group which labeled by “F”, respectively.

**Figure. 2:**
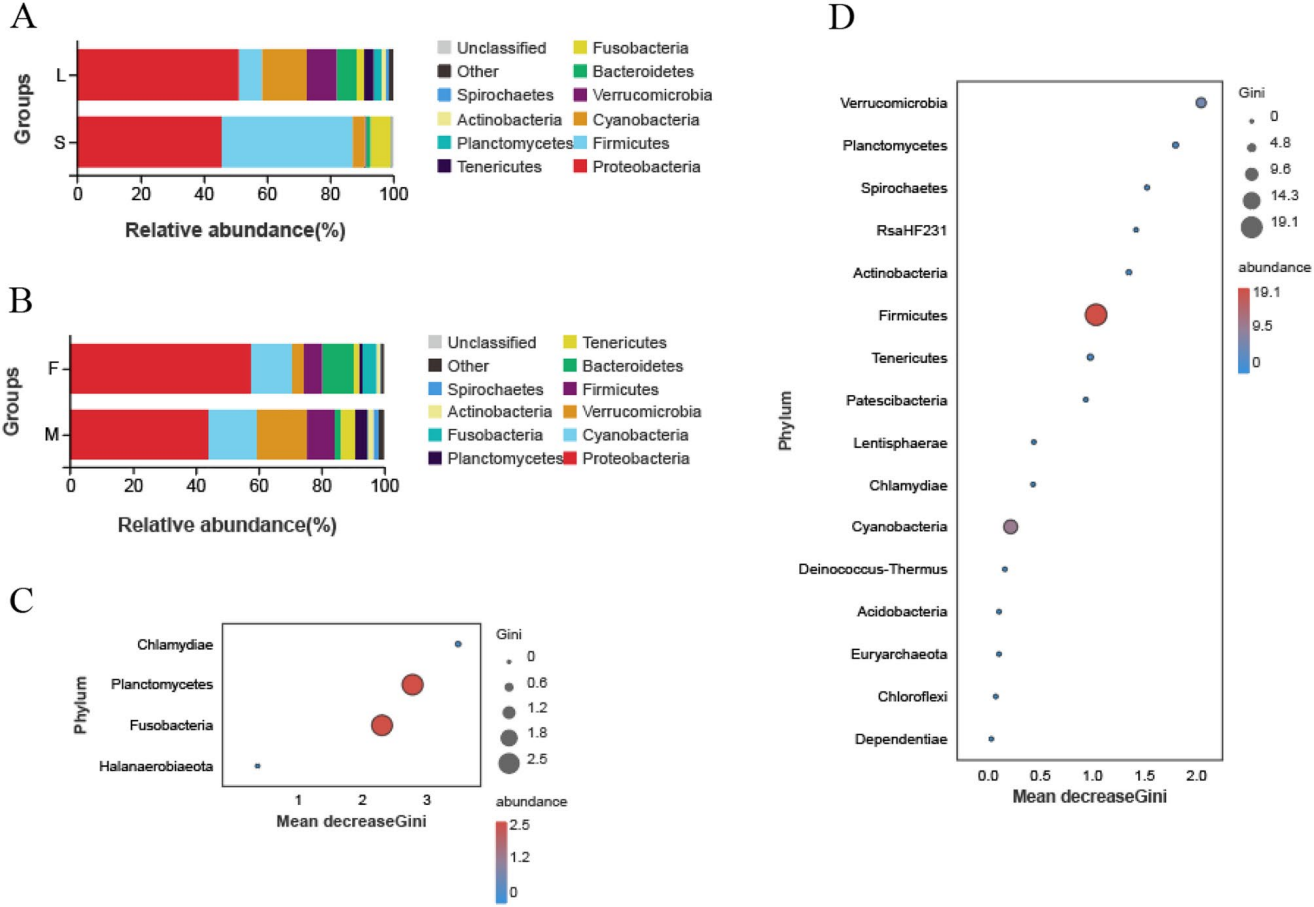
The level distribution of intestinal bacteria phylum differed among the different groups (large vs. small and female vs. male). (**A**) Distribution of intestinal bacteria phylum in large and small groups. (**B**) Distribution of intestinal bacteria phylum in female and male groups. (**C**) Random forest analysis between different gender groups. (**D**) Random forest analysis between different weight groups. The size and color of the bubbles in the figure indicate the abundance of the corresponding species, and the position of the bubbles indicate the size of the index. The greater the effect of the species in distinguishing the two groups, the greater the species Gini index.

There were 10 dominant phyla across all samples, and these represented more than 98.41% of the entire sequence reads. Bacteria within the phylum Proteobacteria were dominant in the intestine of wild *G*. *chilianensis* with 51.01% of the population in the large fish and 45.47% in the small fish, respectively (Fig. 2A). The content of Firmicutes in the small fish (41.52%) was significantly higher than that in the large fish (7.26%) (*p* < 0.05), so this phylum was selected as the most likely indicator species of the groups of large and small fish by random forest analysis (Fig. 2D).

From the perspective of gender grouping, Proteobacteria was the most represented bacteria in females and males (Fig. 2B). The content of Verrucomicrobia in the males (15.93%) was significantly higher than that in the females (3.73%) (*p* < 0.05). However, the content of Bacteroidetes in the females was significantly higher (*p* < 0.05) with 10.01% in the females group, and 2.02% in the males groups. Besides, random forest analysis with the mean decrease Gini index found that Planctomycetes and Fusobacteria each served as indicator bacteria to distinguish between the two genders (Fig. 2C).

### Diversity of intestinal microbiota of *G*. *chilianensis* with different body weights and genders

**Figure. 3:**
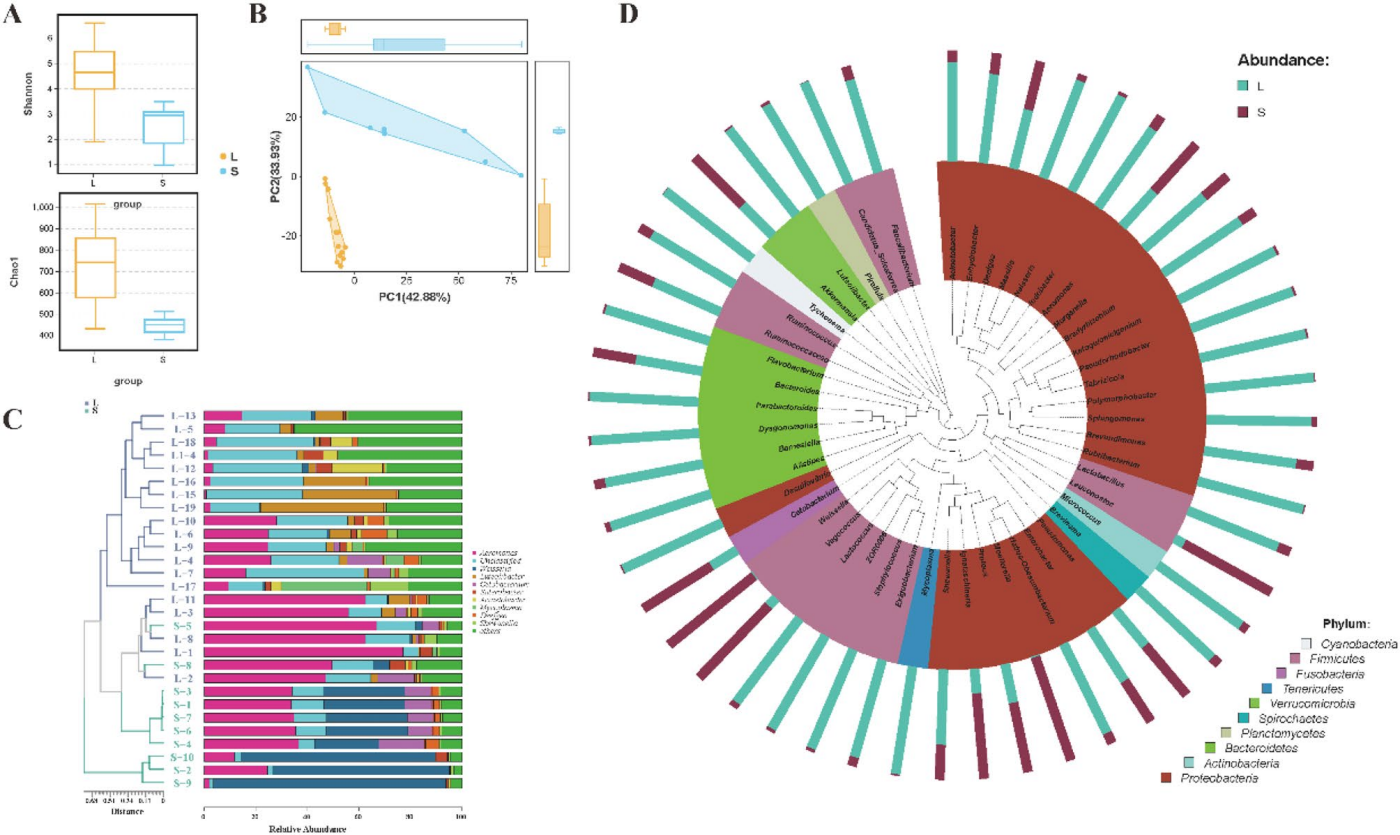
Analysis of intestinal microbial diversity and the genus level difference between different weight groups. (**A**) The difference of the Shannon diversity index and the Chao1 diversity index. (**B**) Principal Component Analysis (PCA) based on OTU abundance information of species. (**C**) Unweighted pair group method with arithmetic mean (UPGMA) cluster tree based on the weighted UniFrac distance. (**D**) Phylogenetic relationship of genera horizontal species. The phylogenetic tree was constructed with the representative sequences of the genera horizontal species. The colors of the branches and fan-shaped branches represent their corresponding gates, and the stacking histogram outside the fan ring represents the abundance distribution information of the genus in the different samples.

**Figure 4:**
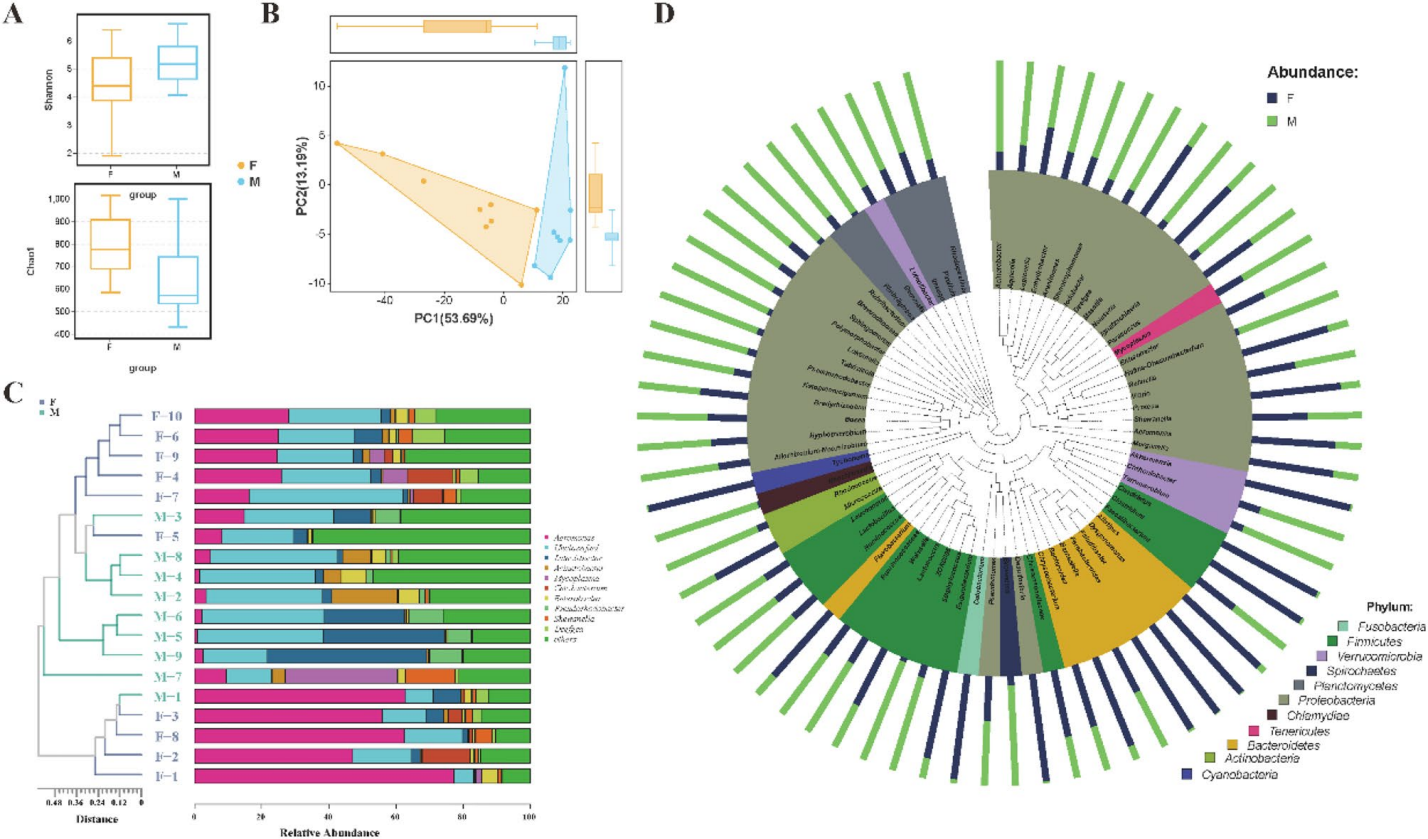
Analysis of intestinal microbial diversity and genus level differences between different gender groups. (**A**) The difference of the Shannon diversity index and Chao1 diversity index. (**B)** Principal Component Analysis (PCA) based on OTU abundance information of species. (**C**) Unweighted pair group method with arithmetic mean (UPGMA) cluster tree based on the weighted UniFrac distance. (**D**) Phylogenetic relationship of genera horizontal species. The phylogenetic tree was constructed with the representative sequences of the genera horizontal species. The colors of the branches and fan-shaped branches represent their corresponding gates, and the stacking histogram outside the fan ring represents the abundance distribution information of the genus in the different samples.

The Shannon index and Chao1 index of the large fish were higher than those of the small fish (Fig. 3A), thereby indicating that the species abundance and evenness degree of the intestinal microbiota of the large fish was higher than that of the small fish. In addition, the Shannon index of the male fish and Chao1 index of the female fish were higher in the sex comparison (Fig. 4A). The closer the samples were to each other, the more similar their microbiome structures were. Principal Component Analysis (PCA) based on the OTU abundance information of species found that samples within each group were closer to each other (Figs. 3B and 4B). Similarly, the Unweighted Pair Group Method with Arithmetic Mean (UPGMA) clustering analysis showed that the large fish, small fish, female fish, and male fish clustered in their respective branches within a small range (Figs. 3C and 4C).

In all the samples, there were 8 genera with relative abundance greater than 1%, which were *Aeromonas*, *Weissella*, *Luteolibacter*, *Cetobacterium*, *Eterobacter*, *Acinetobacter*, *Mycoplasma*, *Deefgea* and *Shewanella* (Fig. 3C). Based on the relative abundance and difference analysis of the genera with average relative abundance greater than 1% in the sample, *Luteolibacter* was mainly enriched in the large fish, while *Weissella* and *Cetobacterium* were mainly enriched in the small fish (Fig. 3C). Additionally, the populations of *G*. *chilianensis*, *Leuconostoc*, *Pseudomonas*, *Moellerella*, *Proteus*, *Ignatzschineria*, *Vagococcus*, *Akkermansia*, and *Aeromonas* in the small fish were significantly higher than those in the large fish, while most of the other bacteria were significantly fewer in number in the small fish compared to the large fish (Fig. 3D). For gender grouping, there were significant differences in two genera, namely *Aeromonas* in female fish and *Luteolibacter* in male fish, respectively (Fig. 4C). Besides, most bacteria (of Fusobacteria, Bacteroidetes, and Cyanobacteria) were significantly greater in number in the females than in the males (Fig. 4D).

### The functional prediction of *G*. *chilianensis* intestinal microbiota differed by body weight and gender

**Figure 5:**
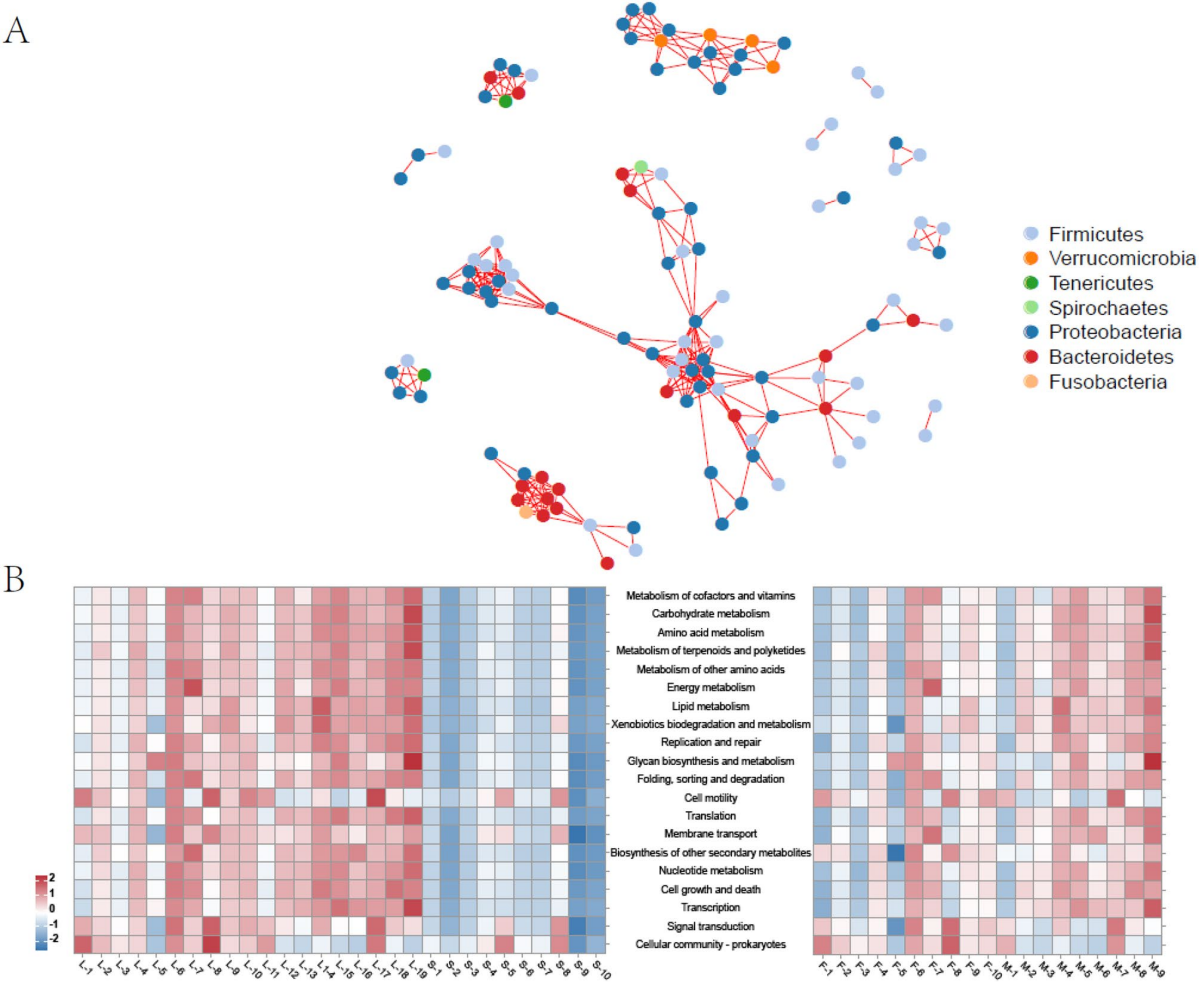
Functional prediction of intestinal microbiota (large vs. small and female vs. male). (**A**) Network diagram of the microbial community structure. (**B**) Heatmaps of functional abundance for different samples.

According to the interaction analysis of bacteria, most bacteria were related to each other. Firmicutes and Proteobacteria are important bacteria in *G*. *chilianensis* and can coexist with many other microorganisms (Fig. 5A). Functional analysis of the microbiota showed that the differentially expressed microbiota observed in *G*. *chilianensis* with different body weight and gender was mainly attributable to metabolic function. To further understand the differential changes in intestinal microbiota under different body weights and gender, we conducted a prediction of bacterial function and explored whether different body weights and gender would affect the function of intestinal microbiota. In the heavier fish, there were 20 upregulated pathways. The functional analysis of microbial communities in the two different gender groups showed that there were 3 upregulated pathways (cell motility, signal transduction and cellular community-prokaryotes) and 11 downregulated pathways (Metabolism of cofactors and vitamins, Metabolism of cofactors and vitamins, and Amino acid metabolism, among other pathways) in the female group (Fig. 5B).

## Discussion

In terms of the Alpha diversity index, the Shannon index and the Simpson index of large individuals were significantly higher than those of small individuals, while those of male individuals were higher than female individuals. The results showed that the richness and evenness of intestinal microbiota increased with age and the flora became more stable. These results are consistent with those fingings for yellow catfish and grass carp^9,10^. The changes in the richness and evenness of intestinal microbiota may be caused by changes in the feeding habits of *G. chilianensis* as they age^12^. *G. chilianensis* is a kind of omnivorous fish with animal preference. In the larval stage, it mainly feeds on diatoms, cladocerans and other plankton. In the adult stage, it feeds more on benthos bait, and sometimes also eats young fish. In addition, the higher diversity index in large individuals may be related to strong swimming ability, but further research is needed to prove this. The differences in the intestinal microbiota between the sexes may be caused by the effects of sex-related hormones^13,14^.

Our study indicated that in the gut microbiome of *G. chilianensis*, the dominant flora of different genders and weights were relatively consistent, and included Proteobacteria, Firmicutes, and Bacteroidetes. At present, these three phyla have been dominant microbiome in the intestinal tracts of many marine and freshwater fish species^15–17^. The similar bacterial taxa in the intestinal microbiota of various fish species suggests that these bacteria are beneficial to the host and would contribute to digestion, nutrient absorption, and immune responses^4^. Noted that the Proteobacteria is the highest in relative abundance among these three phyla, belonging to the resident bacteria in the intestinal tracts of many animal species. The abundance change of content of Proteobacteria would results in the imbalances of intestinal microbiota. The proportion of Proteobacteria, which have many conditional pathogenic bacteria, in the intestinal tract is associated with the microecology and stability of the intestinal microbial community structure. Thus, Proteobacteria is identified as a microbiological indicators of intestinal dysbiosis^18,19^. Bacteroidetes and Firmicutes are both highly important for the fermentation of polysaccharides, and there exists a mutually promoting symbiotic relationship between the two. They jointly promote the absorption and storage of energy in the host, and the change in their ratio consequently affects the metabolic potential of intestinal microbiota in the body. In this study, there were more Firmicutes and fewer Bacteroidetes observed in the intestines of the small fish, and these indicate the strong ability to decompose food and promote host energy absorption^20^. Bacterial interaction analysis also found that Firmicutes and Proteobacteria were associated with most bacteria, which was consistent with the results of previous studies ^21,22^.

At the genus level, the dominant bacterial groups of *G. chilianensis* with different weights and genders include *Aeromonas*, *Weissella*, and *Enterobacter*, which are all normal intestinal microbiota ^23^. *Aeromonas* was the genus with the greatest variation in intestinal abundance among the different groups of *G. chilianensis*. They are widely distributed in nature, and some strains of this genus can efficiently produce amylase in fish to promote intestinal digestion^24^, while others may play a pathogenic role by causing enteritis, sepsis, and other diseases in both humans and animals^25^. In this study, the abundance of *Aeromonas* in the intestines of the small fish was higher than that of the large fish, and their abundance in females was higher than that of males. Whether such strains mainly promote nutrient absorption or cause disease in the body requires further study. *Weissella* are lactic acid bacteria that are ubiquitous in nature and can widely colonize the host intestinal tract^26,27^. The functions of *Weissella* including synthesize dextran, fructan, and other oligosaccharides. These functions of *Weissella* can promote the absorption of trace elements and reduce gastrointestinal discomfort, thus promoting the proliferation of *Bifidobacterium*^28^. The abundance of *Weissella* in the intestinal tract of small individuals was found to be significantly higher than that of large individuals, likely so that nutrients can be better absorbed to confer a faster growth rate^29^. The abundance of *Enterobacter* may be affected by the source of protein consumed^30^. Some bacteria of this genus are conducive to metabolic activities, while some are potential opportunistic pathogens^31^. Whether there are differences in the abundance of *Enterobacter* in the gut caused by different protein sources in different sex individuals remains to be investigated^32^.

The phylogenetic relationships and UPGMA clustering analysis showed differences in the composition and structure of the intestinal microbiota between different body weights and genders, which was similar to those reported to be observed in aquatic animals such as *Coreius guichenoti*^33^. UPGMA clustering analysis showed that individuals with different body weights and sex clustered on their respective branches within a small range, which may be caused by the differences in the environment for different sampling sites within the same river^34^. The results of functional prediction showed that the intestinal microbiota functions of different body weights and genders were highly similar, and the dominant functions mainly included the metabolism of cofactors and vitamins, carbohydrate metabolism, and amino acid metabolism. However, there were some differences observed in the relative abundance of various functions between body weights and sex. Interestingly, Sugita et al. (1982) found that the normal intestinal microbiota of Mozambican tilapia (*Oreochromis mossambicus*) was established 20–60 days after hatching, and speculated that this establishment was related to the development of intestinal structure and function^35^. *G. chilianensis* primarily feed on other animals in the juvenile stage, while the adult fish are omnivorous^36^. Zooplankton, phytoplankton, and aquatic insects comprise the main sources of food. In order to adapt to this change in feeding habits, the intestinal microbiota of *G. chilianensis* concurrently changes in structure and function with the development of the digestive system.

## Methods

### Animal materials

**Figure 6:**
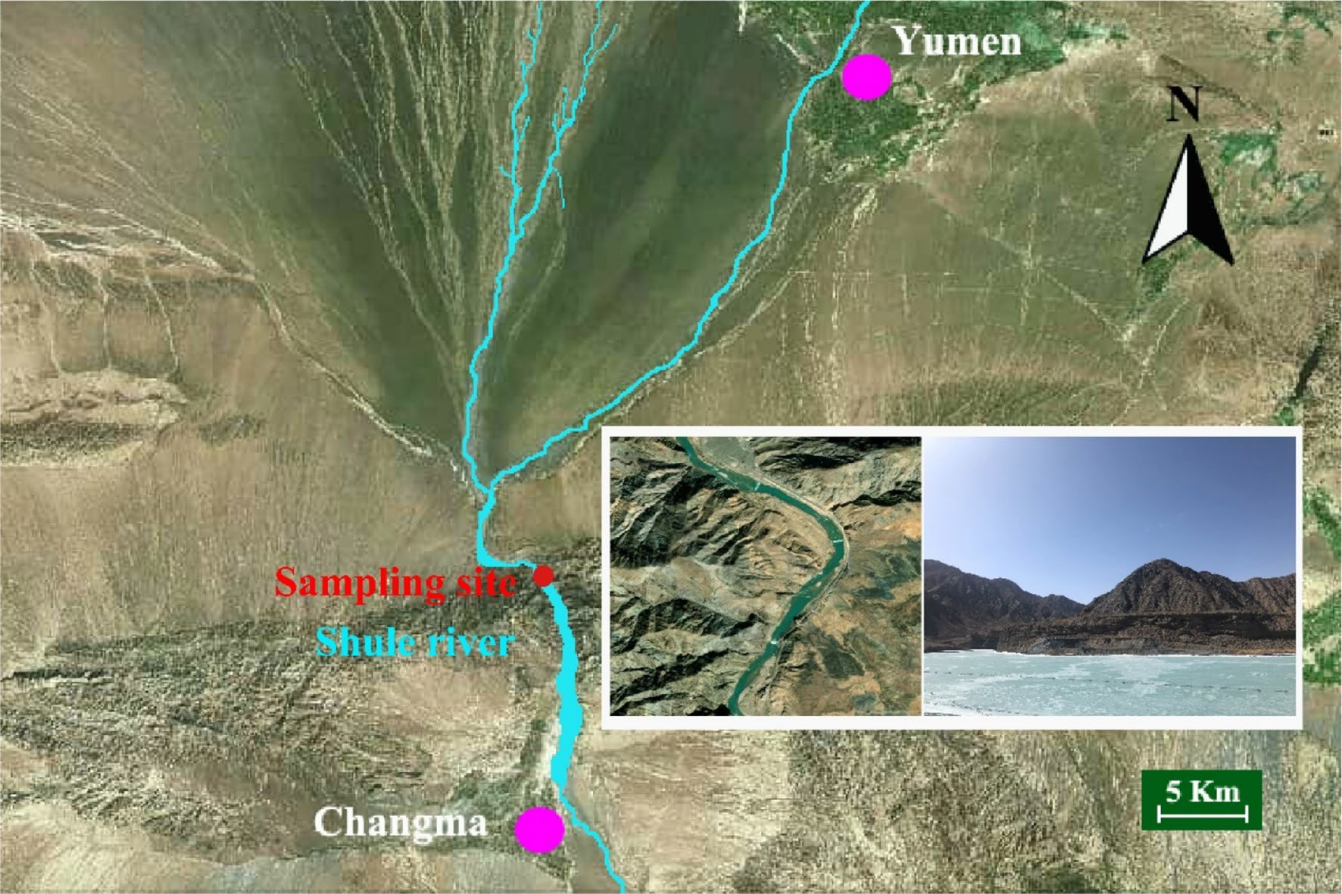
Sample collection sites in the Gansu, China. The spatial pattern of Shule River was acquired by Aowei map with the version of Vip193 (www.gpsov.com). The city of Changma and Yumen were marked by purple circles, and sampling site was marked by red circle. The subgraphs in sampling sites are high-resolution image of geomorphic feature by Aowei map and actual photo, which was made in April 2021, respectively.

All the test fish were collected in Shule River, Gansu Province, China, and the sampling site (96°48′18′′ E, 39°59′23′′ N) and growth environment were shown in Fig. 6. Fish samples were collected using gill nets (mesh: 5 × 5 cm) and ground bamboo cages (mesh: 1 × 1 cm) in April 2021. All fish were placed in oxygen-filled boxes, transported to the lab on ice, and measured for body length and weight. Fish samples that have reached sexual maturity were differentiated according to gonadal development, while fish samples that have not reached sexual maturity are unable to distinguish their gender. All were anesthetized with tricaine mesylate (MS-222) (Sigma-Aldrich, Beijing, China). The surface was sterilized with 75% ethanol before dissecting the entire intestine of the fish using sterile tools (scissors and tweezers). A similar weight (approximately 0.2 g) of intestinal contents was collected respectively from the foregut, midgut and hindgut of each fish and mixed into a single sample (approximately 0.6 g per fish). The individual intestinal contents were homogenized by brief vertexing.

### DNA extraction

Microbial DNA was extracted using the HiPure Soil DNA Kit or HiPure StoolDNA Kit (Magen, Guangzhou, China) according to the manufacturer’s protocols.

### PCR amplification

The 16S rDNA target region of the ribosomal RNA gene was amplified by PCR Refer to the primers in the study of Guo et al.^37^. The resulting amplicons were extracted from the 2% agarose gels and purified using the AxyPrep DNA Gel Extraction Kit (Axygen Biosciences, Union City, CA, U.S.) according to the manufacturer’s instructions, and then quantified using the ABI StepOnePlus Real-Time PCR System (Life Technologies, Foster City, USA). The purified amplicons were pooled in equimolar and pair-end sequenced (PE250) on an Illumina platform according to the standard protocols.

### Statistical analysis

The representative OTU or ASV sequences were classified into organisms by a naive Bayesian model using the RDP classifier (version 2.2) based on the SILVA database (version 132) with the confidence threshold value of 0.8. The abundance statistics of each taxonomy were visualized using Krona (version 2.6)^38–40^. The stacked bar plot of the community composition was visualized in R using the ggplot2 package (version 2.2.1).

Between-group Venn analysis was performed in R using the VennDiagram package (version 1.6.16) and the UpSet plot was created in R using the UpSetR package (version1.3.3) to identify both unique and common species, OTUs, or ASVs^41,42^. The species comparison between groups was calculated by Welch’s t-test and the Wilcoxon rank test in R using the Vegan package (version 2.5.3). The species comparison among groups was computed by Tukey’s HSD test and the Kruskal-Wallis H test in R using the Vegan package (version 2.5.3)^43^. Chao1, ACE, Shannon, Simpson, Good’s coverage, and Pielou’s evenness index were calculated in QIIME [9] (version 1.9.1). The OTU/ASVrarefaction curve and rank abundance curves were plotted in R using the ggplot2 package (version 2.2.1). The alpha index comparison between groups was calculated by Welch's t-test and the Wilcoxon rank test in R using the Vegan package (version 2.5.3).

Sequence alignment was performed using Muscle (version 3.8.31) and the phylogenetic tree was constructed using FastTree (version 2.1), then the weighted and unweighted UniFrac distance matrix was generated by GuniFrac package (version 1.0) in R. Principal component analysis (PCA) was performed in R using the Vegan package (version 2.5.3)^44–46^. The analysis of functional differences between groups was calculated by Welch's t-test, the Wilcoxon rank test, the Kruskal-Wallis H test, and Tukey’s HSD test in R using the Vegan package (version 2.5.3).

### Ethical approval

All animal handling procedures were approved by the Animal Care and Use Committee of the Fisheries Research Institute, Sichuan Academy of Agricultural Sciences (Chengdu, China), following the recommendations in the ARRIVE guidelines, under permit number 20210307001-5. At the same time, all methods were carried out in accordance with relevant guidelines and regulations.

## Conclusion

In conclusion, our research provides the first detailed description of the microbiome structure of *G. chilianensis* under field conditions and demarcated between different genders and body weights. Alpha diversity analysis based on the metrics used in our study revealed that the large fish exhibited a higher intestinal microbiota richness and evenness compared to the small fish, which were also greater for males compared to females. The dominant bacterial groups of different genders and weights were relatively consistent. At the phylum level, Proteobacteria, Firmicutes and Bacteroidetes were dominant, while at the genus level, *Aeromonas*, *Weissella*, and *Enterobacter* were dominant. Both the variables of weight and sex had significant effects on microbial community structure. However, the clustering results showed that both sexes and weights clustered together in small areas, which may be caused by environmental differences in sampling locations. The difference between the different weights may be due to the ontogeny influence on the structure of the intestinal microbiota, and the difference between the sexes may be the result of the secretion of hormones. However, the results of this study should be treated with caution. It cannot be ruled out that environmental differences between sampling sites have an impact on intestinal microbiota structure. Future interspecific studies of individuals from different geographic areas and habitat types are needed to precisely define the role of these factors in shaping gut microbial composition. This study increases the understanding of the diversity of the fish intestinal microbial ecosystem, and provides both a scientific basis and theoretical guidance for the screening of intestinal probiotics to assist in the growth process and the study of probiotic preparations in the aquaculture industry.

## Data Availability

The raw sequence data reported in this paper have been deposited in the Genome Sequence Archive^47^ in National Genomics Data Center^48^, China National Center for Bioinformation / Beijing Institute of Genomics, Chinese Academy of Sciences (GSA: CRA009117) that are publicly accessible at https://bigd.big.ac.cn/gsa/browse/CRA009117.
